# Long-term outcome and eligibility of radiofrequency ablation for hepatocellular carcinoma over 3.0 cm in diameter

**DOI:** 10.1038/s41598-023-43516-w

**Published:** 2023-09-28

**Authors:** Takashi Tanaka, Kazuhide Takata, Takashi Miyayama, Kumiko Shibata, Hiromi Fukuda, Ryo Yamauchi, Atsushi Fukunaga, Keiji Yokoyama, Satoshi Shakado, Shotaro Sakisaka, Fumihito Hirai

**Affiliations:** Department of Gastroenterology and Medicine, Faculty of Medicine, Fukuoka University, 7-45-1, Nanakuma, Jonan-Ku, Fukuoka, 814-0180 Japan

**Keywords:** Gastroenterology, Hepatology, Oncology, Cancer

## Abstract

Percutaneous radiofrequency ablation (RFA) is effective for the treatment of small hepatocellular carcinoma (HCC) with a diameter ≤ 3.0 cm. The present study aimed to elucidate the prognostic factors and clarify the indication of treatment for RFA outcomes in patients with HCC with a diameter > 3.0 cm. Among 2188 patients with HCC who underwent RFA, 100 patients with HCC with a diameter > 3.0 cm were enrolled in this study between August, 2000 and August, 2021. We analyzed local therapeutic efficacy, long-term outcomes, and prognostic factors in patients with HCC with a diameter > 3.0 cm. Among all patients, 77 patients achieved complete ablation in one session. There were no treatment-related deaths or major complications. Local tumor recurrence occurred in 48% (n = 48) of the patients, and distant tumor recurrence occurred in 82% (n = 82) of the patients during the study period. The survival rates at 1-, 3-, 5-, 10-, and 15- years were 93.0%, 66.0%, 40.0%, 15.5%, and 10.2%, respectively. Cox proportional hazards regression analysis confirmed that distant tumor recurrence, Child–Pugh class B, and pre-ablation des-γ-carboxy prothrombin (DCP) levels ≥ 200 mAU/mL were independent unfavorable prognostic factors with a hazard ratio of 3.34 (95% CI, 1.57–7.11; P = 0.002), 2.43 (95% CI, 1.35–4.37; P = 0.003), and 1.83 (95% CI, 1.14–2.93; P = 0.012), respectively. In conclusion, patients with HCC with a diameter > 3.0 cm with Child–Pugh class A and DCP levels < 200 mAU/mL might be eligible for RFA treatment.

## Introduction

Radiofrequency ablation (RFA) is widely performed for the treatment of small hepatocellular carcinoma (HCC) as a curative treatment owing to safety, reasonable cost, and applicability as a minimally invasive technique. Additionally, clinical management guidelines consider RFA as an effective treatment modality for early-stage HCC ^1,2^. However, in some cases, good prognosis has been achieved with RFA even for large HCC with a diameter > 3.0 cm. Several studies have demonstrated that RFA is a potential curative treatment modality for HCC with a diameter > 3.0 cm ^3,4^. Recently, with the advent of new devices, multipolar RFA, that can cause cauterization up to 6 cm in one session, and newer generation microwave ablation systems have been found to achieve treatment success for large HCC ^5–10^. However, only a few studies have elucidated the factors that contribute to the prognosis of the patients with HCC with a diameter > 3.0 cm who were underwent RFA. We started RFA therapy for hepatic tumors at our institution in August 2000; we have encountered more than 1,800 treatment cases, and have used RFA for HCC with a diameter > 3.0 cm. It is noteworthy that several patients who underwent RFA for HCC with a diameter > 3.0 cm achieved good prognosis. The present study aimed to retrospectively analyze the outcome of RFA for HCC with a diameter > 3.0 cm and to elucidate the factors that can predict good prognosis among these patients and reveal the eligibility of RFA treatment for the patients with HCC with a diameter > 3.0 cm.

## Patients and methods

### Study design

This retrospective study was conducted between August 2000 and August 2021 at the Department of Gastroenterology and Medicine, Fukuoka University Hospital, Fukuoka, Japan. The study was approved by the Institutional Review Board (approval no. H21-08–003) and the procedures were conducted in accordance with the principles of Declaration of Helsinki. Written informed consent was obtained from all patients before RFA. All patients underwent routine physical examination and laboratory tests before RFA. Contrast-enhanced dynamic computed tomography (CT) scan and/or magnetic resonance imaging (MRI) were performed within one month prior to RFA. Based on the American Association for the Study of Liver Diseases practice guidelines for HCC management, HCC was diagnosed when the lesion displayed hypervascularity in the arterial phase and washout in the portal venous or delayed phase on dynamic CT and/or MRI^1^.

### Endpoints

The primary endpoint was overall survival (OS) and the secondary end point was progression-free survival (PFS). Additionally, we elucidated the prognostic factors associated with OS and PFS for patients who underwent RFA for the treatment of HCC with a diameter > 3.0 cm using multivariate analysis.

### Selection criteria for RFA

The selection criteria for RFA were as follows: (1) age ≥ 20 years, (2) all HCCs were able to detect by ultrasonography and at least one sized < 7.0 cm, (3) total number of tumors less than four, (4) patients with good liver function less than well-compensated cirrhosis, (5) correctable platelet count more than 50 × 10^3^/mL, (6) prothrombin time-international normalized ratio < 1.5, and (7) patients deemed unsuitable for or refused surgical resection. Patients with major vessel invasion and/or extrahepatic metastases were excluded from this study.

### RFA procedure

Percutaneous RFA was performed by physicians with ≥ 5 years of experience in RFA of liver tumors at our institute. We used a monopolar RFA system that involves the insertion of a 17-gauge internally cooled electrode with a 20- or 30-mm-long exposed tip (Cool-tip; Radionics, Burlington, MA, USA), and two types of multi-tined expandable RFA electrodes: a 17-gauge, 15-cm-long RTC LeVeen probe (Boston Scientific Corporation, Marlborough, MA, USA) and 14-gauge, 15- or 20-cm-long RITA model 90 (RITA Medical Systems, Mountain View, CA, USA). A multipolar RFA system with bipolar internally cooled electrodes (Celon Lab Power, Olympus Corporation, Tokyo, Japan) was available from April 2014. Radiofrequency energy was delivered via a generator using an impedance-based control algorithm, according to the manufacturer’s instructions for each device. Transcatheter arterial chemoembolization (TACE) was performed within 1–2 weeks before RFA for tumors with a diameter > 3.0 cm, multinodular tumors, and/or those located near the hepatic vessels. However, not all patients who met these criteria received TACE before RFA because of poor hepatic function, contraindication for iodinated contrast medium because of allergy or thyroid disease, renal dysfunction, and refusal of the patient. In all cases, the procedure was performed percutaneously under US guidance and local anesthesia. Artificial ascites was induced, when required, to improve the US window and decrease thermal injury to the adjacent diaphragm, lungs, heart, gall bladder, stomach, and colon.

### Follow-up

The radiological response was assessed within 1 week after RFA using contrast-enhanced dynamic CT or MRI. A tumor was considered completely ablated if no nodular or irregular enhancement adjacent to the ablative zone was visible in the arterial phase and if an ablative zone margin ≥ 5 mm from the edge of the tumor was observed in the portal phase.

Physicians examined the patients at 4 weeks after RFA, and liver function tests and tumor markers were evaluated once every 3 months. After the eradication of HCC, tumor recurrence, including local recurrence, in early stage was assessed using contrast-enhanced dynamic CT or MRI every 3 months to detect early-stage tumor, after assessing the therapeutic effects of RFA.

### Statistical analysis

OS and PFS rates were analyzed using the Kaplan–Meier method, and differences between the curves were assessed using the log-rank test. Independent predictive factors associated with OS and PFS were identified using multivariate Cox proportional hazards regression analysis. The following potential risk factors for OS and local tumor recurrence after RFA were evaluated: age, sex, Child–Pugh classification, tumor size, naïve HCC, levels of HCC-specific tumor markers such as alpha-fetoprotein (AFP) and des-gamma-carboxy prothrombin (DCP), and additional TACE before RFA. Statistical analyses were conducted using JMP software for Windows, version 14.2.0; (SAS Institute, Cary, NC, USA). Difference with P value < 0.05 were considered statistically significant.

### Ethical Standard

Approval for this retrospective study was obtained from the local ethical review board (approval no. H21-08-003) of Fukuoka University Hospital. Written informed consent was obtained from patients included in this study.

## Results

### Background of patients and tumors

**Figure 1:**
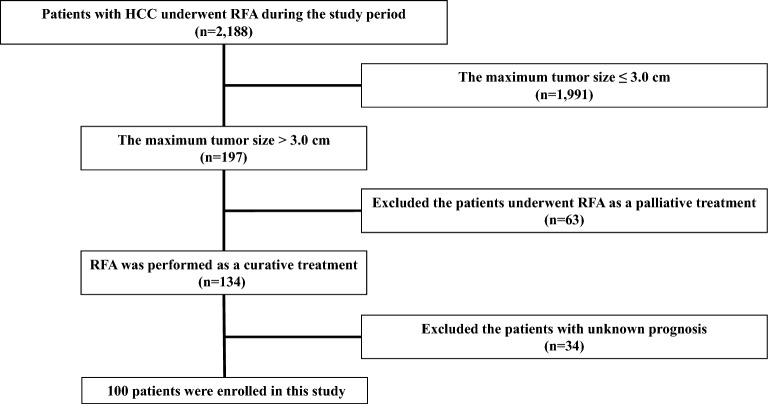
Patient selection process and the study groups.

Among 2,188 patients with HCC who underwent RFA during the study period, 100 patients with HCC with a diameter > 3.0 cm were enrolled in this study (Fig. 1). The baseline characteristics of the patients are presented in Table 1. The median age of patients (65 men and 35 women) was 71.5 years (range 48–88 years). The Child–Pugh class was A (n = 78) and B (n = 22) and 81 patients were associated with viral hepatitis (hepatitis B: n = 5, hepatitis C: n = 76). Median tumor size was 3.5 cm in diameter (range: 3.1–6.2 cm), and the number of tumors was 1, 2, 3, and 4 in 56, 21, 16 and 7 patients, respectively. In all, 53 and 47 patients were classified into the early- and intermediate-stage categories according to the Barcelona Clinic Liver Cancer (BCLC) staging system.

**Table 1:** The baseline characteristics of patients.

Features	Values
Age (years)*	71.5 (48, 88)
Sex*	
Male	65 (65)
Female	35 (35)
Etiology*	
HBV	5 (5)
HCV	76 (76)
NBNC	19 (19)
Child Pugh classification*	
A	78 (78)
B	22 (22)
Naïve HCC*	
Yes	59 (59)
No	41 (41)
Size of the tumor (cm) †	3.5 (3.1, 6.2)
Number of tumors*	
1	56 (56)
2	21 (21)
3	16 (16)
4	7 (7)
BCLC staging system	
Early-stage	53 (53)
Intermediate-stage	47 (47)
Combined with TACE*	
Yes	63 (63)
No	37 (37)
AFP (ng/mL) †	31.5 (1.6, 3061)
DCP (mAU/mL) †	82 (7.8, 12,502)

*HBV* Hepatitis B virus; *HCV* Hepatitis C virus; *NBNC* Non B non C; alcoholic, nonalcoholic steat hepatitis, autoimmune hepatitis; *BCLC* Barcelona Clinic Liver Cancer; *TACE* Transcatheter arterial chemoembolization; *RFA* Radiofrequency ablation; *AFP* Alpha-fetoprotein; *DCP* Des-gamma-carboxy prothrombin.

*Number of the Patients (%).

^†^Median (minimum, Maximum).

### Result of RFA

Among the four types of RFA devices, Cool-tip was used in 82 patients (82%), RTC LeVeen probe in 7 patients (7%), Celon Lab Power in 7 patients (7%) and RITA model 90 in 4 patients (4%). There were no treatment-related deaths or major complications in our study population. RFA was performed in one session in 77 patients (77%) and ≥ 2 sessions in 23 patients (23%), and all patients acquired sufficient ablative margin. Local tumor recurrence occurred in 48 patients (48%), with a median time of 43 months (range 1–159 months) for local tumor recurrence. Distant tumor recurrence occurred in 82 patients (82%), with a median time of 12 months (range 1–136 months).

### Long-term outcome

**Figure 2:**
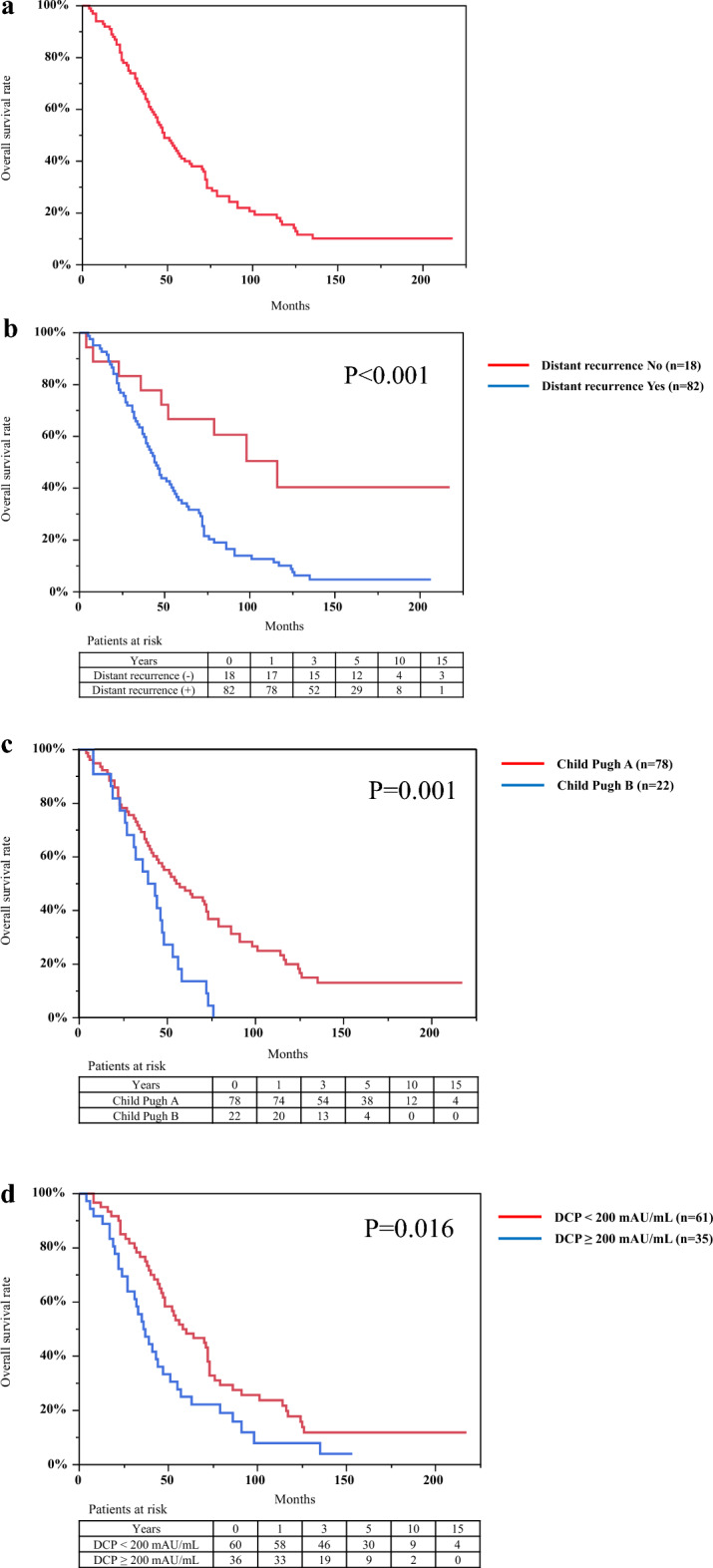
Kaplan–Meier curves for overall survival of patients with hepatocellular carcinoma with a diameter > 3.0 cm who were treated with radiofrequency ablation. (**a**) Cumulative overall survival rates at 1-, 3-, 5-, 10-, and 15- years were 93%, 66%, 40%, 15.5%, and 10.2%, respectively. (**b**) Difference in overall survival rates for distant tumor recurrence Yes (n = 82) vs. No (n = 18), (**c**) Child–Pugh class A (n = 78) vs. B (n = 22), (**d**) des-gamma-carboxy prothrombin levels < 200 mAU/mL (n = 61) vs. ≥ 200 mAU/mL (n = 35) were statistically significant based on the log-rank test (P < 0.001, P = 0.001, P = 0.016, respectively).

We evaluated long-term outcomes, including OS and PFS. Median OS was 48 months (95% confidence interval [CI], 40–60 months). Cumulative OS rates at 1-, 3-, 5-, 10-, and 15- years were 93%, 66%, 40%, 15.5%, and 10.2%, respectively (Fig. 2a). OS rates for distant tumor recurrence Yes (n = 82) vs. No (n = 18), Child–Pugh class A (n = 78) vs. class B (n = 22), and DCP levels < 200 mAU/mL (n = 61) vs. ≥ 200 mAU/mL (n = 35) are shown in Fig. 2b-d, respectively. The differences between the aforementioned values were statistically significant based on the log-rank test (distant tumor recurrence, P < 0.001; Child–Pugh class, P = 0.001; Levels of DCP, P = 0.010). The OS rates between BCLC early- and intermediate-stage groups were not statistically different in the log-rank test (P = 0.061) (Supplementary Fig. S1). Multivariate Cox proportional hazards regression analysis revealed that predictive factors associated with OS were distant tumor recurrence (hazard ratio [HR], 3.34; 95% CI, 1.57–7.11; P = 0.002), Child–Pugh class B (HR, 2.43; 95% CI, 1.35–4.37; P = 0.003), and DCP levels ≥ 200 mAU/mL (HR, 1.83; 95% CI, 1.14–2.93; P = 0.012) (Table 2).

**Table 2:** Prognostic factors about radiofrequency ablation for the patients with HCC > 3.0 cm in diameter.

Characteristic	Univariate analysis		Multi variate analysis	
	HR (95% C.I)*	P value†	HR (95% C.I)*	P value†
Age (years) ≥ 70	1.13 (0.68–1.71)	0.564	1.22 (0.78–1.92)	0.290
Sex (Male)	1.59 (0.74–2.03)	0.050	1.53 (0.92–2.54)	0.099
Etiology; Viral Hepatitis (Yes)	1.22 (0.61–2.04)	0.678	1.24 (0.65–2.40)	0.514
Child Pugh classification B	2.29 (1.37–3.82)	0.002	2.43 (1.35–4.37)	0.003
Naïve HCC (Yes)	0.76 (0.49–1.16)	0.205	0.73 (0.44–1.20)	0.209
Tumor size (cm) < 4.0	1.04 (0.63–1.70)	0.871	0.92 (0.53–1.58)	0.752
Single tumor (Yes)	0.70 (0.45–1.07)	0.095	0.75 (0.48–1.17)	0.206
Combined with TACE (Yes)	0.80 (0.52–1.23)	0.974	1.22 (0.75–1.97)	0.426
APF (ng/mL) ≥ 200	0.76 (0.45–1.29)	0.307	1.38 (0.74–2.56)	0.308
DCP (mAU/mL) ≥ 200	1.70 (1.09–2.65)	0.018	1.83 (1.14–2.93)	0.012
Distant tumor recurrence (Yes)	3.08 (1.53–6.19)	0.002	3.34 (1.57–7.11)	0.002

*HR* Hazard ratio; *CI* Confidence interval; *HCC* Hepatocellular carcinoma; *TACE* Transcatheter arterial chemoembolization; *AFP* Alpha-fetoprotein; *DCP* Des-gamma-carboxy prothrombin.

*Relative risks were calculated by comparing classes with Cox regression analysis.

^†^P-value were obtained by using Cox regression analysis.

**Figure 3:**
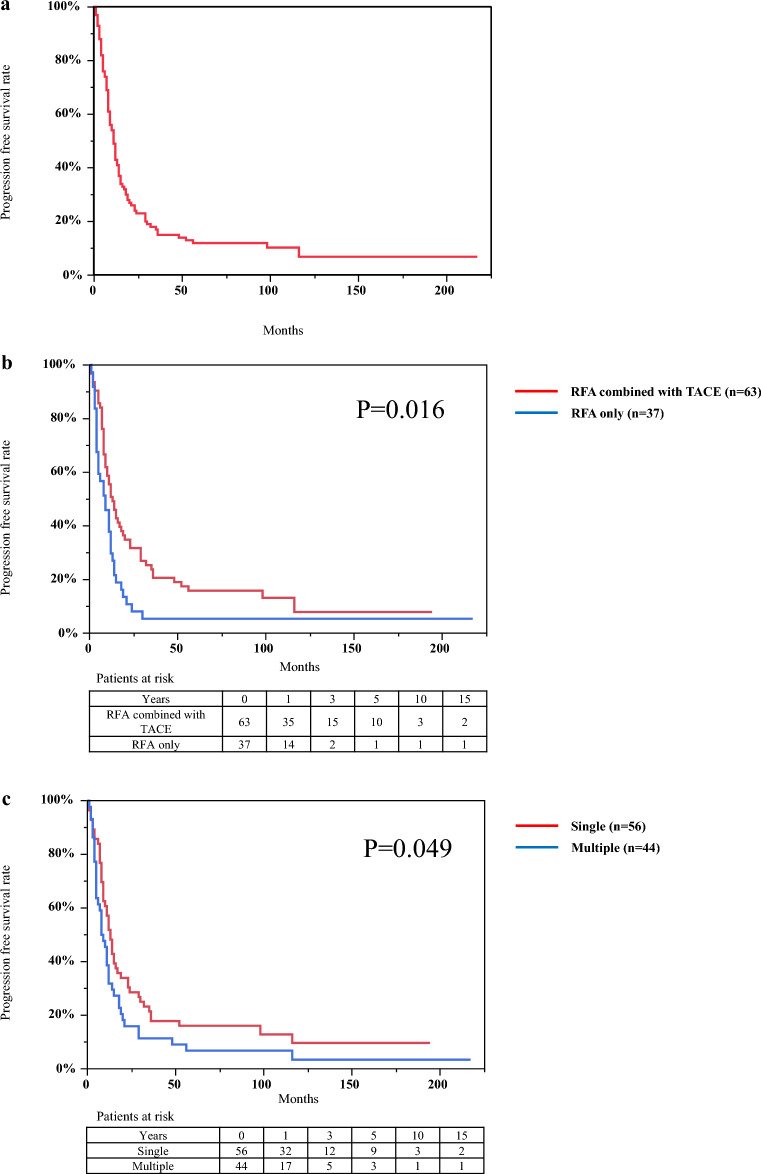
Kaplan–Meier curves for progression-free survival of patients with hepatocellular carcinoma with a diameter > 3.0 cm who were treated with radiofrequency ablation. (**a**) Cumulative progression-free survival rates at 1-, 3-, 5-, 10-, and 15- years were 43%, 15%, 12%, 6.7%, and 6.7%, respectively. (**b**) Difference in progression-free survival rates for combination with TACE; Yes (n = 63) vs. No (n = 37) and (**c**) the number of tumors; single (n = 56) vs. multiple (n = 44) were statistically significant based on the log-rank test (P = 0.016 and P = 0.049, respectively).

The median PFS was 11 months (95% CI, 9–14 months). Cumulative PFS rates at 1-, 3-, 5-, 10-, and 15- years were 43%, 15%, 12%, 6.7%, and 6.7%, respectively (Fig. 3a). PFS rates in RFA combined with TACE Yes (n = 63) vs. No (n = 37) and the number of tumors single (n = 56) vs. multiple (n = 44) are shown in Fig. 3b,c, respectively. The differences between the aforementioned values were statistically significant based on the log-rank test (RFA combined with TACE, P = 0.016; the number of tumors, P = 0.049). The PFS rates between BCLC early- and intermediate-stage groups were not statistically different in the log-rank test (P = 0.051) (Supplementary Fig. S2). Multivariate Cox proportional hazards regression analysis revealed that predictive factors associated with PFS was RFA combined with TACE (HR, 0.60; 95% CI, 0.38–0.96; P = 0.033) (Table 3).

**Table 3:** The factors associated progression free survival with radiofrequency ablation for the patients with HCC > 3.0 cm in diameter.

Characteristic	Univariate analysis		Multi variate analysis	
	HR (95% C.I)*	P value†	HR (95% C.I)*	P value†
Age (years) ≥ 70	1.06 (0.70–1.60)	0.780	1.00 (0.64–1.56)	0.992
Sex (Male)	1.34 (0.86–2.08)	0.197	0.98 (0.61–1.60)	0.952
Etiology; Viral Hepatitis (Yes)	1.11 (0.66–1.89)	0.688	0.80 (0.44–1.47)	0.478
Child Pugh classification B	1.23 (0.76–2.00)	0.405	1.27 (0.73–2.22)	0.397
Naïve HCC (Yes)	0.75 (0.49–1.14)	0.176	0.64 (0.40–1.03)	0.063
Tumor size (cm) < 4.0	1.00 (0.62–1.62)	0.998	0.66 (0.37–1.21)	0.179
Single tumor (Yes)	0.67 (0.44–1.01)	0.058	0.69 (0.44–1.08)	0.104
Combined with TACE (Yes)	0.57 (0.37–0.88)	0.012	0.60 (0.38–0.96)	0.033
APF (ng/mL) ≥ 200	0.64 (0.38–1.08)	0.098	0.71 (0.40–1.28)	0.259
DCP (mAU/mL) ≥ 200	1.30 (0.84–2.00)	0.240	1.23 (0.79–1.92)	0.355

*HR* Hazard ratio; *CI* Confidence interval; *HCC* Hepatocellular carcinoma; *TACE* Transcatheter arterial chemoembolization; *AFP* Alpha-fetoprotein; *DCP* Des-gamma-carboxy prothrombin.

*Relative risks were calculated by comparing classes with Cox proportional hazard regression analysis.

^†^P-value were obtained by using Cox proportional hazard regression analysis.

### Combination of Child–Pugh class and DCP values for OS

**Figure 4:**
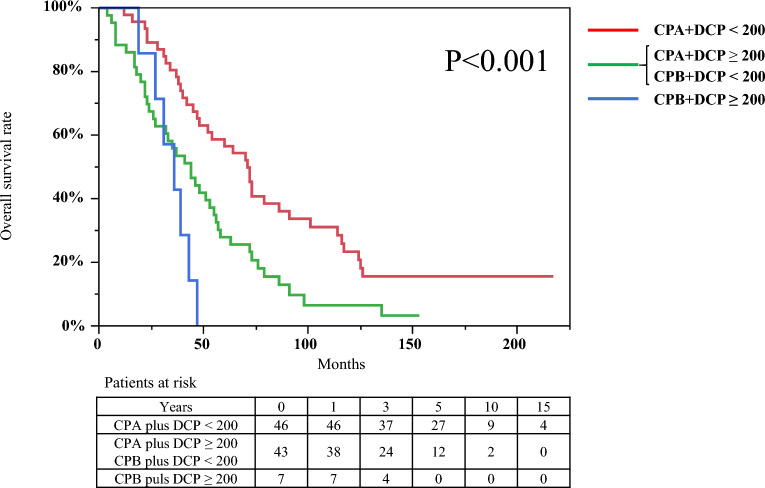
Kaplan–Meier curves for overall survival of patients with hepatocellular carcinoma with a diameter > 3.0 cm based on Child Pugh class and des-gamma-carboxy prothrombin levels. Cumulative overall survival rates for the combination of Child Pugh class A and des-gamma-carboxy prothrombin (DCP) levels < 200 mAU/mL group (n = 46) at 1-, 3-, 5-, 10-, and 15- years were 97.8%, 78.3%, 56.5%, 23.3%, and 15.5%, respectively. Difference in overall survival rates among the three groups, viz., Child Pugh class A and DCP levels < 200 mAU/mL group, Child Pugh class B and DCP levels < 200 mAU/mL or Child Pugh class A and DCP levels ≥ 200 mAU/mL group (n = 43), and Child Pugh class B and DCP levels ≥ 200 mAU/mL group (n = 7), were statistically significant based on the log-rank test (P < 0.001).

We evaluated the significance of two factors associated with OS. Patients were divided into the following three groups: Child Pugh class A and DCP levels < 200 mAU/mL group, n = 46; Child Pugh class B and DCP levels < 200 mAU/mL or Child Pugh class A and DCP levels ≥ 200 mAU/mL group, n = 43; and Child Pugh class B and DCP levels ≥ 200 mAU/mL group, n = 7. We found that OS was significantly different among the three groups (log-rank P < 0.001) (Fig. 4). Median OS of Child Pugh class A and DCP levels < 200 mAU/mL group was 71 months (95% CI, 47–86 months). Cumulative OS rates of Child Pugh class A and DCP levels < 200 mAU/mL group at 1-, 3-, 5-, 10-, and 15- years were 97.8%, 78.3%, 56.5%, 23.3%, and 15.5%, respectively. Median OS of Child Pugh class B and DCP levels ≥ 200 mAU/mL group was 36 months (95% CI, 19–43 months). Cumulative OS rates of Child Pugh class B and DCP levels ≥ 200 mAU/mL group at 1-, 3-, and 5- years were 100%, 42.8%, and 0%, respectively.

## Discussion

This is the first report that the over 20 years long-term outcome of RFA treatment for the patient with HCC in diameter > 3.0 cm. In the present study, some patients with HCC in diameter > 3.0 cm could have a long prognosis underwent RFA. Currently, treating HCC with a diameter > 3.0 cm by nonsurgical methods remains a challenge. According to the current guidelines, surgical resection may be effective in selected patients with a single HCC with a diameter > 3.0 cm; however, owing to chronic liver disease or cirrhosis, less than 20% of patients are eligible for surgery^11–14^. In clinical practice, ablation treatment or TACE is recommended for patients with HCC with a diameter > 3.0 cm who are ineligible for surgery due to decreased cardiopulmonary function or tumor localization that is unsuitable for resection such as porta hepatis. Several studies have reported that RFA combined with TACE has a good treatment effect among patients with HCC with a diameter > 3.0 cm^15–18^. In our study, RFA combined with TACE was not a predictive factor for OS, but it was a predictive factor for PFS, as identified by multivariate Cox proportional hazards regression analysis (HR, 0.60; 95% CI, 0.38–0.96; P = 0.033); therefore, we suggest that RFA combined with TACE may be effective for preventing local and distant tumor recurrence in patients with HCC with a diameter > 3.0 cm.

Although there have been many studies discussing the treatment for HCC with a diameter > 3.0 cm with technical procedural aspects such as additional TACE or novel procedures such as multipolar RFA or newer generation microwave ablation system, there are few studies elucidating factors that contribute to the prognosis and make the patients with HCC with a diameter > 3.0 cm eligible for RFA. Yin et al. elucidated the long-term outcomes of RFA in patients with HCC with a diameter > 3.0 cm and reported that incomplete tumor ablation, recurrent tumors, and pre-ablation AFP levels ≥ 200 ng/mL were independent unfavorable prognostic factors for HCC ranging from 3.0 to 7.0 cm in diameter^3^. Our observation period was longer than that of Yin’s study (21 years vs. 10 years) and to the best of our knowledge, this is the first study based on such a long observational period.

In the present study, the predictive factors associated with OS were distant tumor recurrence (HR, 3.34; 95% CI, 1.57–7.11; P = 0.002), Child–Pugh class B (HR, 2.43; 95% CI, 1.35–4.37; P = 0.003), and DCP levels ≥ 200 mAU/mL (HR, 1.83; 95% CI, 1.14–2.93; P = 0.012) as revealed by multivariate Cox proportional hazards regression analysis. Furthermore, we suggested that patients with HCC with a diameter > 3.0 cm having good liver function (Child–Pugh class A) and DCP levels < 200 mAU/mL were eligible for RFA. DCP, also known as the protein induced by vitamin K absence/antagonist-II, is a form of prothrombin that is specifically produced at high levels by a proportion of HCCs^19–22^. A previous study suggested that DCP could predict the microinvasion of small HCCs measuring < 2.0 cm in diameter^23^. This implies that DCP-positive cases have microvascular invasion, and high DCP levels are predictive of poor prognosis. Several studies have reported that DCP is an important predictive factor for PFS and OS following treatment for HCC^24–28^. Shiina et al. reported that the serum DCP level alone was significantly related to local tumor progression among patients with HCC treated using RFA^27^. Kobayashi et al. reported that high serum DCP levels reflect the biological aggressiveness and progression of HCC, and are predictive of poor prognosis after RFA of HCC ^28^. Furthermore, we previously reported that OS rates of patients with HCC with a diameter < 3 cm (n = 472) following RFA at 3-, 5-, 7-, and 10- years were 79.4%, 55.6%, 36.3%, and 27.4%, respectively^29^. In the present study, OS rates of patients with HCC with a diameter > 3 cm having Child Pugh class A and DCP levels < 200 mAU/mL following RFA at 3-, 5-, 7- and 10- years were 78.3%, 56.5%, 38.5%, and 23.3%, respectively. These rates are similar to those of patients with HCC with a diameter < 3 cm. Therefore, we suggest that patients with HCC with a diameter > 3 cm having Child Pugh class A and DCP levels < 200 mAU/mL are eligible for RFA.

Several studies have reported the beneficial effect of RFA in patients with BCLC classification based intermediate-stage HCC^15,16,30–34^. Azuma et al. examined 59 patients with BCLC intermediate- stage HCC and concluded that additional RFA of nodules treated insufficiently by TACE was effective^30^. Yin et al. examined 211patients and reported an additional beneficial effect of RFA on TACE-treated patients^34^. Indeed, most of the reports suggest an additional benefit of RFA for the treatment of BCLC intermediate- stage HCC, which had been previously treated with TACE. Nouso et al. reported that the effect of RFA was comparable to that of TACE alone, regardless of whether RFA was administered alone or in combination with TACE^35^. Furthermore, several previous studies have suggested that TACE is recommended for patients with BCLC intermediate- stage HCC, but suboptimal response rates have resulted in TACE being used as a palliative measure^36–38^; however, diverse therapies for BCLC intermediate- stage HCC are now available. In addition to TACE, which is recommended for the treatment of patients with intermediate- stage HCC in a classical BCLC algorithm, local ablation therapy, sorafenib, and liver transplantation have been suggested as alternatives in the new recommendation^38^. In the present study, the log-rank test to compare OS and PFS rates between BCLC early- and intermediate-stage groups revealed no significant differences (OS, P = 0.061, PFS, P = 0.051). Therefore, RFA might be suitable for patients with BCLC intermediate- stage HCC with a diameter > 3.0 cm with Child–Pugh class A and DCP levels < 200 mAU/mL.

The local tumor recurrence rate (48%) observed in this study was higher than that reported in a previous representative study^27^. This discrepancy is potentially attributable to several reasons. One, the ablation technique for larger-sized HCC over 3 cm in diameter was difficult to perform, and the ablative zone may have been an insufficient depending on the tumor location. Second, the local tumor recurrence rate might have increased because of the long observation period of 20 years.

The present study has some limitations. First, the present study was a single-center study with a small sample size; additional studies involving more patients treated with RFA are needed to further investigate the predictive factors. Second, this study was retrospective and not randomized with respect to the RFA treatment, and comparisons of RFA with surgical resection and TACE were not performed. Third, there was a selection bias for patients who underwent RFA. The patients in this study could not undergo resection or TACE because of compromised liver function or other complications, such as cardiovascular disease, pulmonary disease, and neurological problems. Patient selection was biased toward those among whom the number of tumors, tumor size, and tumor localization were favorable for local therapy. Forth, there were 76 patients with hepatitis C in our study, and most of them could not undergo antiviral therapy using direct antiviral agents (DAA) during the study period before 2014. Several studies have reported that antiviral treatment for hepatitis C prevents HCC development significantly^39–41^. Hosaka et al. reported that long-term treatment with nucleoside analogues may reduce the incidence of HCC in hepatitis B infected patients^42^. Furthermore, antiviral treatment for patients with hepatitis C may reportedly distant tumor recurrence^43^; thus, treatment with DAAs may have led to improved OS and PFS rates in the present study. However, the prognosis of patients with hepatitis B and C who underwent antiviral treatment after RFA could not be determined because most patients had participated in the present study before DAA were developed for treatment.

In conclusion, we elucidated the predictive factors for good prognosis in patients with HCC with a diameter > 3.0 cm who underwent RFA. Distant tumor recurrence, Child–Pugh class B, and DCP levels ≥ 200 mAU/mL were associated with poor prognosis in this population. Therefore, patients with HCC with a diameter > 3.0 cm with good liver function (Child–Pugh class A) and DCP levels < 200 mAU/mL might be eligible for RFA treatment.

### Supplementary Information

Supplementary Information 1.

Supplementary Information 2.

## Data Availability

The datasets used and analyzed during the current study available from the corresponding author on reasonable request.

## Abbreviations

HCC: Hepatocellular carcinoma
RFA: Radiofrequency ablation
HBV: Hepatitis B virus
HCV: Hepatitis C virus
HR: Hazard ratio
TACE: Transcatheter arterial chemoembolization
AFP: Alpha-fetoprotein
DCP: Des-gamma-carboxy prothrombin
BCLC: Barcelona Clinic Liver Cancer
DAA: Direct antiviral agents
