# Supplementary material for: Long-term outcome and eligibility of radiofrequency ablation for hepatocellular carcinoma over 3.0 cm in diameter
Source: Sci Rep. 2023 Sep 28;13:16286. doi: 10.1038/s41598-023-43516-w (PMC10539460; doi:10.1038/s41598-023-43516-w)
Supplement: Supplementary file 1 — Supplementary Information 1. [file 41598_2023_43516_MOESM1_ESM.pptx]

## Slide 1
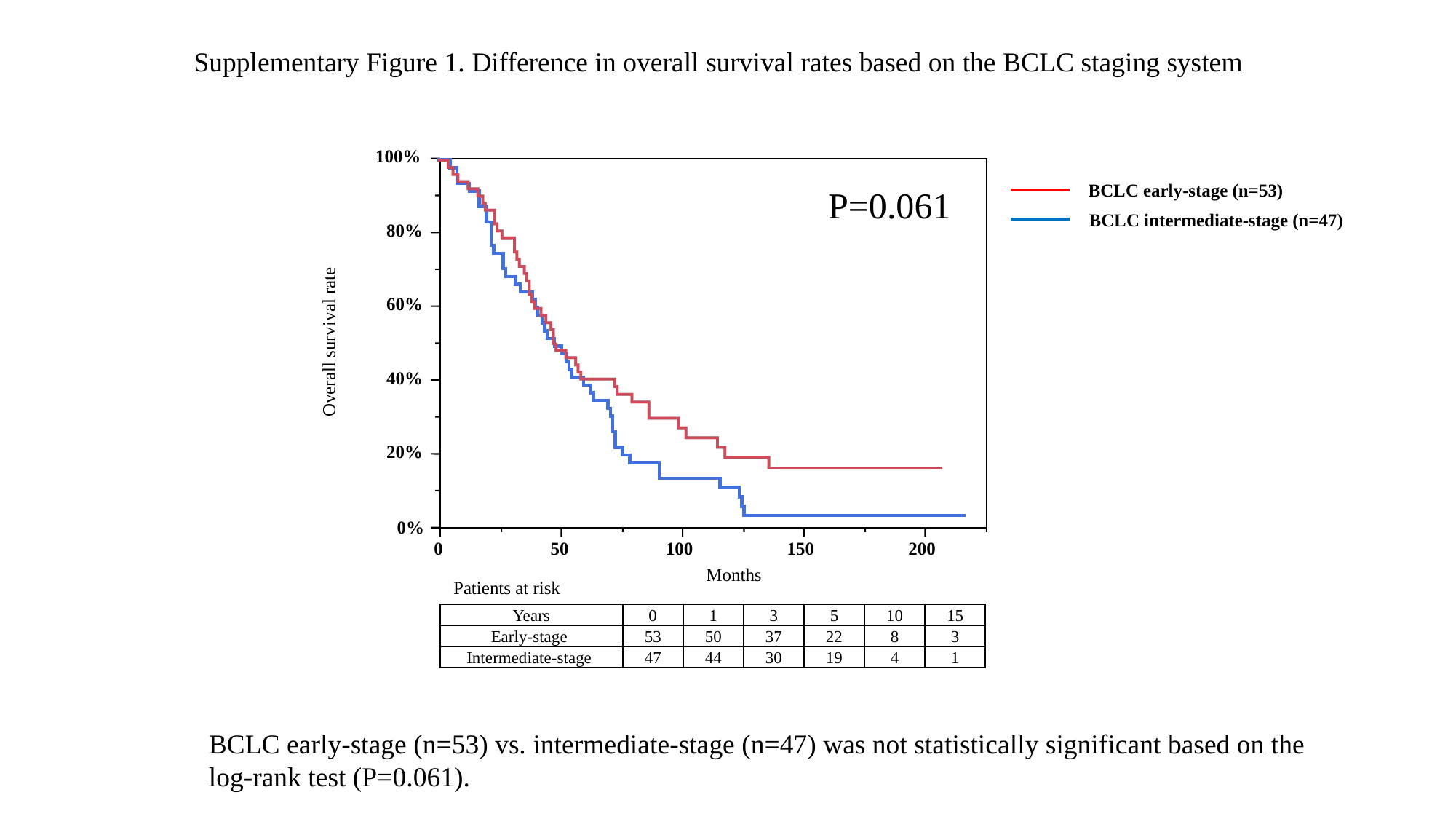

Supplementary Figure 1. Difference in overall survival rates based on the BCLC staging system
100%
BCLC early-stage (n=53)
BCLC intermediate-stage (n=47)
P=0.061
80%
Overall survival rate
60%
40%
20%
0%
0
50
100
150
200
Months
Patients at risk
| Years | 0 | 1 | 3 | 5 | 10 | 15 |
| --- | --- | --- | --- | --- | --- | --- |
| Early-stage | 53 | 50 | 37 | 22 | 8 | 3 |
| Intermediate-stage | 47 | 44 | 30 | 19 | 4 | 1 |
BCLC early-stage (n=53) vs. intermediate-stage (n=47) was not statistically significant based on the log-rank test (P=0.061).
