# Supplementary material for: Long-term outcome and eligibility of radiofrequency ablation for hepatocellular carcinoma over 3.0 cm in diameter
Source: Sci Rep. 2023 Sep 28;13:16286. doi: 10.1038/s41598-023-43516-w (PMC10539460; doi:10.1038/s41598-023-43516-w)
Supplement: Supplementary file 2 — Supplementary Information 2. [file 41598_2023_43516_MOESM2_ESM.pptx]

## Slide 1
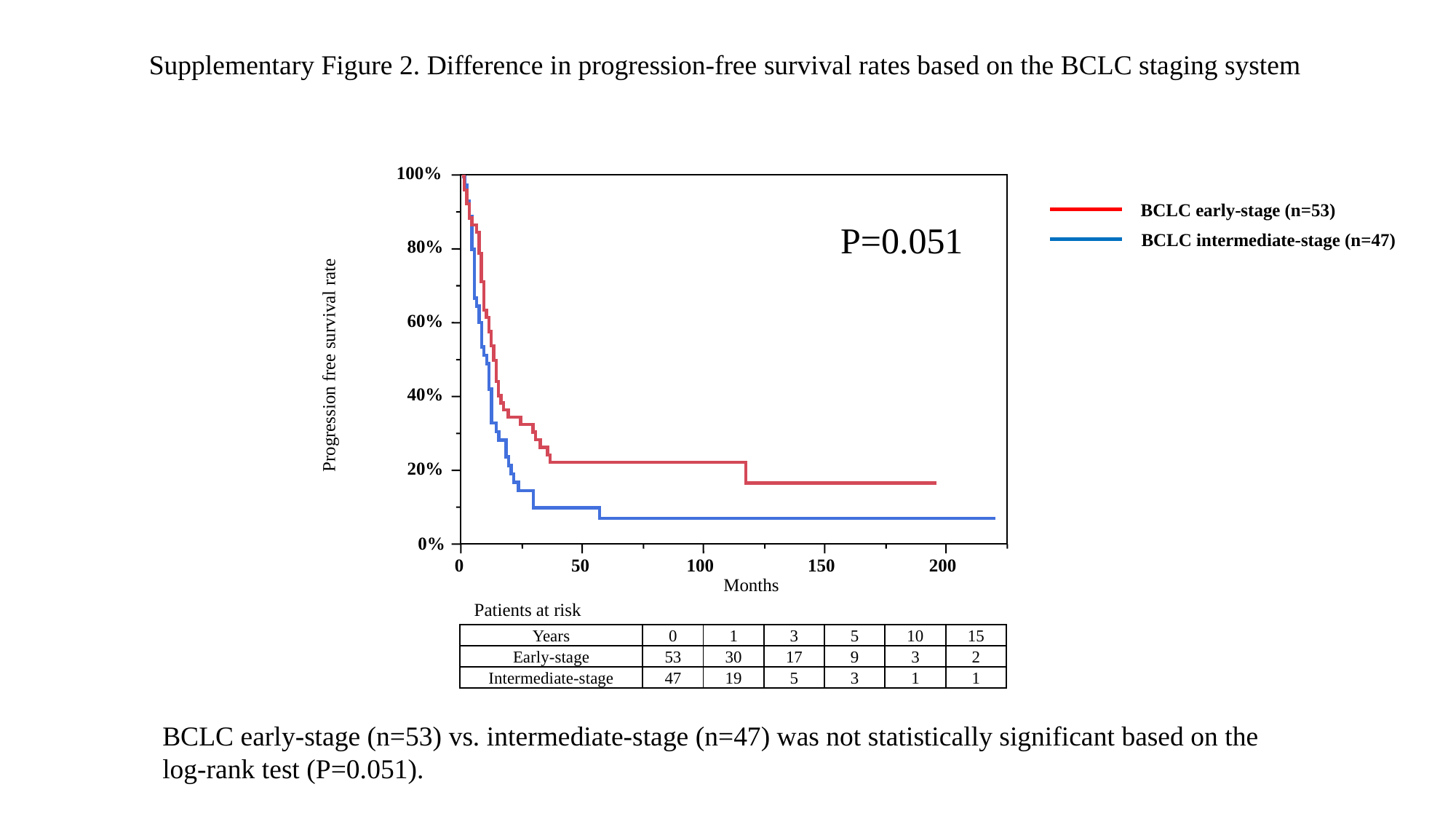

Supplementary Figure 2. Difference in progression-free survival rates based on the BCLC staging system
100%
BCLC early-stage (n=53)
BCLC intermediate-stage (n=47)
P=0.051
80%
Progression free survival rate
60%
40%
20%
0%
0
50
100
150
200
Months
Patients at risk
| Years | 0 | 1 | 3 | 5 | 10 | 15 |
| --- | --- | --- | --- | --- | --- | --- |
| Early-stage | 53 | 30 | 17 | 9 | 3 | 2 |
| Intermediate-stage | 47 | 19 | 5 | 3 | 1 | 1 |
BCLC early-stage (n=53) vs. intermediate-stage (n=47) was not statistically significant based on the log-rank test (P=0.051).
